# Gene Expression Quantification of Toll like Receptors 2, 4 and Co-molecules in Human Glioblastoma Cell Line (U87-MG): Toward a New *In vitro* Model of Inflammation

**Published:** 2011

**Authors:** Amir Mohammad Malvandi, Jalil Mehrzad, Masoud Saleh Moghaddam

**Affiliations:** 1 *Department of Biochemistry, Faculty of Science, Payam Noor University of Mashhad, Iran*; 2 *Department of Stem cells and Developmental Biology, Royan Institute for Stem cell Biology and Technology, ACECR, Tehran, Iran*; 3 *Department of Pathobiology, Faculty of Veterinary Medicine, Ferdowsi University of Mashhad, Iran*

**Keywords:** Human glioblastoma cell line (U87-MG), Innate immunity, Quantitative RT-PCR Toll-like receptors

## Abstract

**Objective(s):**

Pattern recognition receptors (PRRs) are the main part in the innate immune response. Human glioblastoma cell line (U87-MG) is an established adherent cell line model of this common cancer; due to genetic variations between individuals it is likely more suitable for investigating molecular aspects of innate immunity. Therefore, we undertook a novel characterization of the immune phenotype of U87-MG toward establishing a base for future researches.

**Materials and Methods:**

In this study, U87-MG cells where cultured in a normal condition, to investigate levels of toll-like receptor 2 (TLR2), TLR4, myeloid differentiation factor-88 (MyD88) and CD14 transcripts expression in these cells. Both RT-PCR and qPCR were applied to detect and quantify the expression levels of these genes in U87-MG cells and compare them to their levels in the peripheral blood mononuclear cells (PBMC) of healthy individuals, as a common reference.

**Results:**

Expression level of TLR2 and TLR4 are not significantly different in U87-MG cells in comparison to PBMC. Also, expression levels of MyD88 and CD14 in U87-MG cells are significantly lower than their levels in PBMC. Furthermore, expression levels of MyD88 and CD14 in both PBMC and U87-MG are significantly lower than TLR2 and TLR4 transcripts.

**Conclusion:**

The data reveal expression of TLR4, CD14, MyD88 and TLR2 genes in U87-MG cell line, for the first time. Expression detection of these genes in human glioblastoma cell line might have a potential for diagnosis of inflammatory mechanisms in immune mediated disorders of *in vitro* models of human brain inflammatory disease.

## Introduction

Glioblastoma is the most common malignant primary tumor of the human brain (1). In spite of combined treatment modalities including surgery, chemotherapy and radiotherapy, the prognosis of glioblastoma remains very poor, with a median survival below 15 months (2), urging the indispensable need to develop new therapeutic approaches, such as immunotherapy.

The toll-like receptor (TLR) family is one of the best-characterized pattern recognition receptors (PRRs) and is responsible for sensing pathogens both outside the cell and in intracellular endosomes and lysosomes. TLR stimulation can initiates a signal transduction pathway via MyD88 which leads to secretion of pro-inflammatory cytokines such as Interleukin 1 (IL-1), IL-6 and TNF- through activation of nuclear factor kappa B (NF-B) (3).

There is a list of ligands for TLRs which are best-characterized: TLR4 could recognize lipopolysaccharide (LPS; endotoxin) from Gram-negative bacteria cell wall; stimulation of TLR1, TLR2 and TLR6 could be occur after binding bacterial lipoproteins, lipotechoic acid and fungal zymosan as their specific ligands, respectively; In addition, there are highly growing numbers of self-molecules (endogenous) as novel candidate ligands of TLRs. Specifically for TLR2 and TLR4, these include heat shock proteins such as HSP60, HSP70, HSPB8 and α-crystallin A chain (4-10), crystalline shape of uric acids (11), high mobility group box 1 (HMGB1) (12), surfactant protein A (13), and several components of the extracellular matrix such as fibronectin (14), fibrinogen (15), biglycan (16), heparan sulphate (17), hyaluronan breakdown fragments (18) and oligosaccharides of hyaluronan (19). Moreover, it has been well accepted that TLR4 needs some co-receptors such as CD14, for being functional at an optimum state. CD14 receptors act as a carrier linking LPS to the cell surface (20). It is thought that there are two forms of the CD14 receptors. The first one is detectable at the surface of myeloid cells (mCD14) acting as a glycosyl-phosphatidyl-inositol (GPI)-anchored membrane glycoproteins. The second form is serum soluble (sCD14) and does not reveal GPI properties, although it might bind LPS to stimulate cells independent of mCD14, such as epithelial or endothelial cells and smooth muscle cells of the vascular system (21). Recently, there are increasing evidences to support the role of these intensively studied TLRs and their cooperators in molecular pathogenesis of several diseases such as cancer progression (22).

Chronic inflammation is a risk factor for the development of cancer, in part due to the generation of reactive oxygen and nitrogen species (23). Progression of the cancer is often associated with a generalized immunosuppression of the host. Recent evidence suggests that chronic inflammation may be responsible for cooperation of toll-like receptors in cancer (24). However, the source of chronic inflammation in cancer, in the absence of infection, remains incompletely understood. 

In this study, U87-MG cell line were chosen as an *in vitro* model to detect and quantify the expression levels of innate immunity receptor genes transcripts in comparison to PBMC of healthy individuals as a reference model control of these genes expression, to make a clue in probable roles of TLRs in chorionic inflammatory conditions may involve in cancer progressions and future therapeutic approaches. 

## Materials and Methods

***Cell culture and PBMC isolation***

Human glioblastoma cell line (U87-MG, ATCC), a generous gift from Dr Michel Monner’s lab from University of Bordeaux, France, was cultured in 10% FCS/DMEM-high glucose (Gibco) supplemented with 1% penicillin/streptomycin (10000 u/l Gibco), HEPES buffer and L-glutamine (Gibco) at 5% CO_2_ in 95% humidity. 

In order to PBMC isolation, Blood samples were collected by sterile venipunctures from 3 healthy young men. PBMC were isolated from heparinized blood by lympholyte^®^-H (Cederlane laboratories Ltd., Netherland) through gradient centrifugation. After two times washing with phosphate buffer saline (PBS), cells pellets were used for RNA extraction.

***RNA extraction and cDNA synthesis***

Total RNA was extracted using *TriPure* Isolation Reagent () according to manufacture instruction. After treatment with DNase I (Fermentas), RNA was quantified using NanoDrop 3300 (Thermo scientific, ). One g total RNA (DNase I treated) from each sample was used as template for the reverse transcription reaction. cDNA was synthesized using oligo-dT primers and M-Mulv reverse transcriptase (Fermentas). All the samples were reverse transcribed under the same conditions (70 °C for 5 min, 37 °C 5 min, 42 °C for 1 hr and 70 °C 10 min).

***Reverse transcription PCR (RT-PCR)***

Polymerase chain reactions (PCR) were performed in the same reaction properties for toll like receptor 2, 4, CD14 and GAPDH according to the standard protocols with the primers indicated in Table 1. Briefly, cDNA (200 ng) was reacted with 250 mM dNTPs, 1 x reaction buffer (Fermentas), forward and reverse primers (10 pM) and 0.4 units Taq polymerase in a 25 μl final reaction volume. PCR conditions were as follows, 1 cycle of 94 °C for 4 min followed by 36 cycles of 1 min at 94 °C, 51-56 °C (depending on primers) for 1 min and 72 °C for 1 min followed by 1 cycle at 72 °C for 10 min. Ten μl of each PCR product was electrophoresed on a 2% agarose (Merck) gel in 1x TAE buffer at 84 V for 1 hr and visualized with ethidium bromide under UV light (Biorad).

***Quantitative real-time PCR***

Quantitative PCR (Q-PCR) was carried out using same primers of RT-PCR experiments, designed by others (25,26) and rechecked with Prime3plus software, to human specific toll-like receptors (TLR) 2, 4, CD14 and MyD88 while using GAPDH as an endogenous control (Table 1). Q-PCR was performed by ABsolute™ QPCR SYBR^®^ Green Mix kit (Thermo fisher scientific) according to manufacture instruction on the Swift Spectrum^TM^ 48 Real Time Thermal Cycler PCR machine (Esco Micro Pte. Ltd, ). Samples were heated to 95 °C for 10 min, and then subjected to 44 cycles of amplification by melting at 95 °C and annealing at 51-56 °C (depending on the primers) for 1 min. Experimental samples were run in duplicate with the same concentration of cDNA per reaction. To check the amplicon contamination, each run contained no template controls in triplicate for each probe used. Cycle threshold (C_T_) values were recorded. Data were transformed and relatively compared using the comparative Ct method (27).

***Statistical analysis***

Data are expressed as mean±standard deviation. Statistical differences were determined using one way analysis of variance (ANOVA) with Tukey assay for multiple comparisons. Confidence intervals (CI) are indicated in text for each statistical comparison, otherwise CI is considered as 95% of confidence. All tests were performed using PASW 18 statistical software. 

## Results

***Gene expression detection in U87-MG cell line***

**Figure 1:**
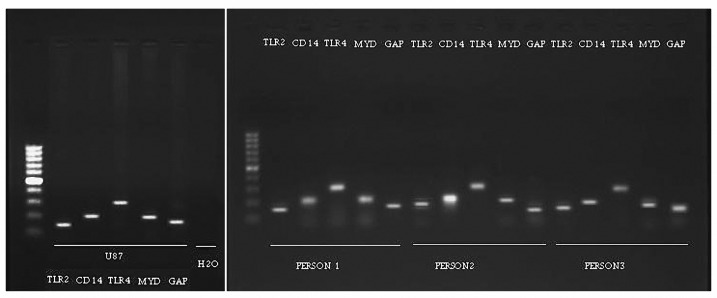
Agarose gel electrophoresis documentation under UV light. Part (a) shows result of RT-PCR experiment for U87-MG cell line which indicated TLR2, CD14, TLR4 and MyD88 genes expression, Non-template control also was performed in favor of PCR reaction quality. Part (b) shows result of the same RT-PCR experiment for different PBMCs isolated from three healthy individuals. GAPDH gene was used as control of cDNA quality for each sample. All reactions were performed together in a same PCR machine at one time.

We used reverse transcription (RT)-PCR to detect the expression of TLR2, 4, CD14 and MyD88 transcripts in U87-MG cell line and PBMC of healthy individuals. After performing several RT-PCRs for separate samples of different passages of U87-MG cell line and samples of PBMC gel electrophoresis were applied to compare results of experiment. According to the results obtained from gel electrophoresis documentation (see Figure 1, a), it is clear that TLR2, 4, CD14 and MyD88 genes are constantly expressed in this cell line. Beside this, according to the control and compare the mentioned results, we performed RT-PCR in a same condition for total RNAs extracted from PBMC of the three different healthy men (Figure 1, b). To qualify the mRNA isolation procedure, GAPDH was used as a reference control in the both experiments.

***Analysis of gene expression levels by qRT-PCR***

**Figure 2.**
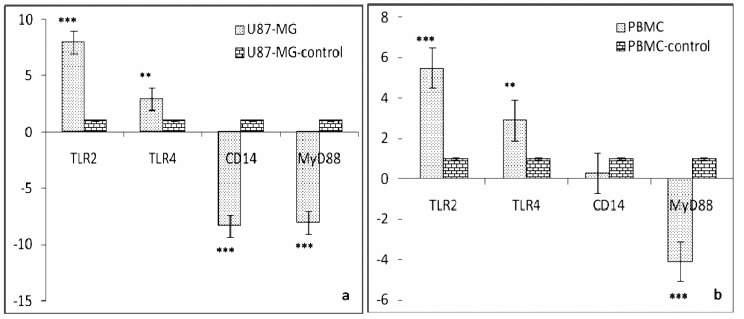
Quantitative real-time PCR analysis of TLR2, TLR4, MyD88 and CD14 in U87-MG cell line (a) and PBMCs of healthy individuals (b). Stars indicate statistically significant difference. Details of statistical analysis are described in the text. Fold changes in each sample were compared to its GAPDH gene expression level (mentioned as control). Data are presented as means±SD.

To quantify the expression level of TLR2, TLR4, CD14, MyD88 and GAPDH (as a baseline control) transcripts in U87-MG cell line and PBMC of healthy human donors, we used relative quantitative RT-PCR. According to the data of this approach (Figure 2), in case of U87-MG cell line; the expression level of TLR2 was the highest and significantly different to TLR4 (*P*< 0.03), CD14 and MyD88 (*P*< 0.001). While the expression level of TLR4 was lower than TLR2, it was significantly higher than both CD14 and MyD88 (*P*< 0.001). However, the expression levels of CD14 (-9.322±2.585) and MyD88 (-8.072±1.575) did not have much difference. 

**Figure 3.**
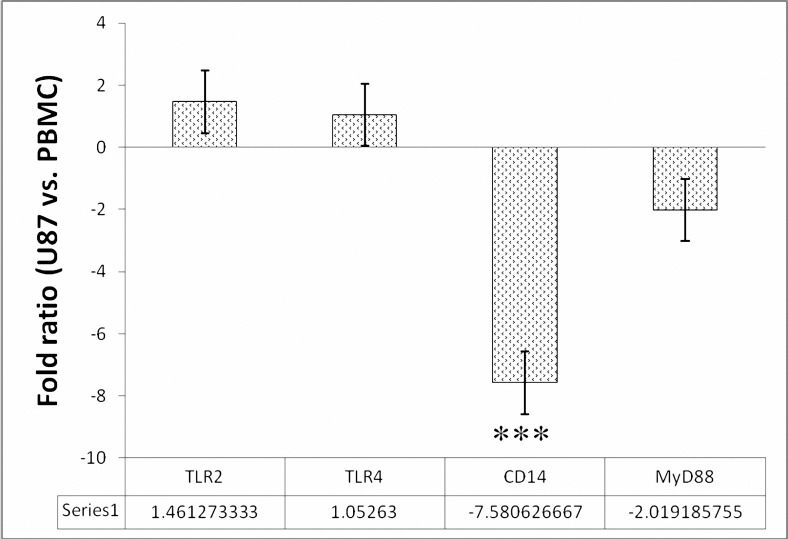
Gene expression fold ratio of U87-MG cell line versus PBMCs of healthy individuals. Ratios are calculated by dividing the qPCR obtained genes expression levels in U87-MG cells into their expression levels (folds) in PBMC. Data in form mean±standard deviation were used for calculations. In the case of MyD88 to visualize a real ratio, because of down-regulation of this gene in both cell types, the ratio is multiply with a minus coefficient (-1) and then presented in the graph. As it shown in the graph the expression level of TLR2 and TLR4 are up-regulated near 1.5 times in U87-MG cell line in comparison to PBMC. In contrast, MyD88 was down-regulated near 2 times in U87-MG cells than PBMC. Interestingly, CD14 was detected near 8 times down-regulated in U87-MG cells less than PBMC and it is significantly different from other ratios (*P*< 0 .001).

In case of PBMC of all three healthy individuals together, gene expression levels showed a similar pattern with U87-MG cell line with slightly variations; the expression level of TLR2 was not significantly higher than TLR4 and CD14, but it had significant difference with MyD88 (*P*< 0.002). Moreover, statistical analysis indicated no significant difference between expression levels of PBMC 

expressed TLR2 (pbmcTLR2) and PBMC expressed TLR4 (pbmcTLR4), PBMC expressed CD14 (pbmcCD14), U87-MG cell line expressed TLR2 (u87TLR2) and U87-MG cell line expressed TLR4 (u87TLR4). On the other hand, calculated ratio of each specific gene expression level in U87-MG cell line versus its level in PBMC, indicated that in U87-MG TLR2, TLR4 genes were expressed about tow fold more than PBMC of healthy individuals, while MyD88 and CD14 were significantly (*P*< 0.001) down-regulated in U87-MG cell line (Figure 3).

**Table 1.** Primer characters were used in qPCR.

Gene	Primer	Sequense (5' -> 3')	Amplicon Size (bp)	References
TLR2	Forward	ATCCTCCAATCAGGCTTCTCT	163	1
	Reverse	ACACCTCTGTAGGTCACTGTTG		
TLR4	Forward	ATATTGACAGGAAACCCCATCCA	300	1
	Reverse	AGAGAGATTGAGTAGGGGCATTT		
CD14	Forward	ACTTGCACTTTCCAGCTTGC	202	1
	Reverse	GCCCAGTCCAGGATTGTCAG		
MyD88	Forward	GACCCCTGGTGCAAGTACC	197	1
	Reverse	AGTAGCTTACAACGCATGACAG		
GAPDH	Forward	GAGCCACATCGCTCAGACAC	150	2
	Reverse	CATGTAGTTGAGGTCAATGAAGG		

## Discussion

In general, it is believed that inflammation can promote/progress carcinogenesis process by multiple mechanisms. These include the anti-apoptotic effect of nuclear factor-κB (NF-κB), induction of the tissue repair response and oxidative damage to DNA (28,29). One of the main controversial points in understanding the association between inflammation and cancer is to identify the triggering events that lead to the inflammatory responses which in turn can promote tumorgenesis (30).

Actually, Inflammation is an inducible response that can be started by several abnormal conditions, such as infection, tissue injury or any alterations in tissue homeostasis. Traditionally, infection is the well understood stimulus of inflammation, after recognition of microbial pathogens by the host innate immune system; a potent inflammatory response will be initiated (31). In this issue, while innate immune responses can be initiated by several types of pattern-recognition receptors (PRRs), the toll-like receptors (TLRs) are the best-characterized (3). 

As our results show, u87TLR4 has upper degree of expression compared to pbmcTLR4. This is supported by other reports in some other cancerous cells (32) such as human colon cancer (33); actually, high expression of this gene indicates a putative role in inflammation-induced tumorgenesis. Thus, it is thought that TLRs would be cooperating in tumor cell growth by direct modulation of cell survival signaling pathways (33). 

We have found also the highest level of gene expression for TLR2 transcript in U87-MG cells. Recent studies have shown that TLRs can also indirectly promote tumor growth by facilitating the creation of an inflammatory microenvironment. For instance, an extracellular matrix proteoglycan which is found in Lewis lung carcinoma (LLC), versican, stimulates TLR2 on macrophages to produce tumor promoting cytokines such as TNF, IL-1b, and IL-6 (34). Beside these, TLRs might provide a condition for tumor cell enabling them to relieve/escape from host surveillance. For example, triggering a cancer cell line form mouse colon carcinoma (M26) with LPS leads to production of IL-6 and consequently inhibits T cells proliferation and NK cell activity (35). Also it was found that TLR2 is able to promote cell proliferation in mouse hepatocarcinoma through JNK and ERK phosphorylation (36). Thus, our findings are confirmed by the mentioned reports in similar conditions. Meanwhile, it is evident that further experiments are needed to find fundamental role of TLR2 in glioblastoma and brain inflammation. 

In downstream TLR signaling pathways it has been found that different adaptor proteins are required for tailored response to each condition/circumstance of cell physiology leading to production of pro-inflammatory cytokines (37) or cell proliferation, including MyD88 dependent pathway which is considered as a core of TLR signaling pathways (22). Once a TLR (except TLR3) is activated by its ligand(s), it is believed that the TLR recruit their specific repertoire of the Toll/Interleukin-1 receptor (TIR)domain adaptors such as MyD88, resulting in activation of a downstream pathway which eventually leads to the activation of NF-B and secretion of pro- inflammatory cytokines or cell proliferation regulation (3). The observed low level of MyD88 transcripts expression in both PBMC and U87-MG cells might be due to the non-infection normal status of cells used in our experiments. Also, the difference between expression levels of MyD88 transcript in PBMC and U87-MG cells was not significant.

A remarkable feature of TLR2 and TLR4 is their ability to cooperate with CD14 on the host cell surface in sensing LPS of Gram negative bacterial infection (3). Modulations of bacterial pattern recognition receptors are important in infection (31). Our data clearly show that U87-MG cell line is CD14 positive. 

Interestingly, its expression level is significantly lower than the levels in PBMC of healthy individuals. It might indicate the functional association of these molecules in innate immunity mechanisms of tumors. 

Adaptive immune responses might be activated by triggered innate immune system (38). This phenomenon is critical in eradication or suppression of tumors. (39). Moreover, in the case of human brain tumors, there is a missing which is a sufficient *in vitro* model to studying these complicated mechanisms. U87-MG is a cancerous cell line isolated form from human brain which is able to produce IL-6 (40) and seems to be useful for this purpose as a suitable *in vitro* model.

## Conclusion

Our data clearly showed the expression of TLR2, TLR4, CD14 and MyD88 transcripts in U87-MG cell line for the first time. According to the well known potentials of this cell line in producing pro-inflammatory cytokines and based on our results, it could be concluded that these cells are a new useful *in vitro *model for researches working on the association of human brain disease inflammation with cancer. Nevertheless, further experimental studies are needed to understand these cell line functional abilities/capacities.
